# Processed lateral root of *Aconitum carmichaelii* Debx.: A review of cardiotonic effects and cardiotoxicity on molecular mechanisms

**DOI:** 10.3389/fphar.2022.1026219

**Published:** 2022-10-17

**Authors:** Jing Zhang, Dan Li, Dan Zhong, Qinmei Zhou, Yanpeng Yin, Jihai Gao, Cheng Peng

**Affiliations:** ^1^ State Key Laboratory Breeding Base of Systematic Research, Development and Utilization of Chinese Medicine Resources, Chengdu University of Traditional Chinese Medicine, Chengdu, China; ^2^ School of Pharmacy, Chengdu University of Traditional Chinese Medicine, Chengdu, China; ^3^ Hospital of Chengdu University of Traditional Chinese Medicine, Chengdu, China

**Keywords:** Fuzi, heart failure, cardiotonic, chemical component, compound formula, cardiotoxicity, molecule mechanism

## Abstract

Fuzi, the lateral root of *A. carmichaelii* Debx., is a typical traditional herbal medicine with both poisonousness and effectiveness, and often used in the treatment of heart failure and other heart diseases. In this review, we searched domestic and foreign literature to sort out the molecular mechanisms of cardiotonic and cardiotoxicity of Fuzi, also including its components. The major bioactive components of Fuzi for cardiotonic are total alkaloids, polysaccharide and the water-soluble alkaloids, with specific mechanisms manifested in the inhibition of myocardial fibrosis, apoptosis and autophagy, and improvement of mitochondrial energy metabolism, which involves RAAS system, PI3K/AKT, JAK/STAT, AMPK/mTOR signaling pathway, etc. Diester-diterpenoid alkaloids in Fuzi can produce cardiotoxic effects by over-activating Na^+^ and Ca^2+^ ion channels, over-activating NLRP3/ASC/caspase-3 inflammatory pathway and mitochondria mediated apoptosis pathway. And three clinically used preparations containing Fuzi are also used as representatives to summarize their cardiac-strengthening molecular mechanisms. To sum up, Fuzi has shown valuable cardiotonic effects due to extensive basic and clinical studies, but its cardiotonic mechanisms have not been systematically sorted out. Therefore, it is a need for deeper investigation in the mechanisms of water-soluble alkaloids with low content but obvious therapeutic effect, as well as polysaccharide.

## Introduction

The pathogenesis of cardiovascular diseases is related to pathological links and processes such as hypertension and arrhythmias, which have become the number one cause of death worldwide due to their high incidence and incurability. Nevertheless, heart failure, the end stage of many heart diseases, mainly manifests as a series of clinical syndromes such as dyspnea and edema, and nowadays has become an issue of concern of people all over the world. According to the data published by the GBD (Global Burden of Disease), there were an estimated 64.3 million people with heart failure all over the world between 1990–2017 (GBD 2017 Disease and Injury Incidence and Prevalence Collaborators, 2018), with 6,70,000 new cases each year (Ambrosy et al., 2014). In developed countries, the overall prevalence of heart failure is in the range of 1%–2%, and in Asia it is between 1% and 1.3%, which is similar to Western countries (Groenewegen et al., 2020).

The treatment of heart failure is usually based on chemical drugs, including diuretics and angiotensin-converting enzyme inhibitors, etc. Although these drugs are more significant in relieving some symptoms, patients may experience serious side effects after long-term use, such as bradycardia and hypotension, which bring poor quality of life to patients (Wang et al., 2017). Traditional Chinese medicine is widely welcomed in the United States, Australia and other developed countries. As an integral part of traditional Chinese medicine, TCM herbs are characterized by “good efficacy, low side effects, and multi-targets,” and have shown reliable efficacy in treating chronic diseases in recent years, so that it is considered as an alternative therapy for heart failure (Jin and Zhang, 2020).

*Aconiti Lateralis* Radix Praeparata, named Fuzi in Chinese, is derived from the lateral roots of *A. carmichaelii* Debx. Compendium of Materia Medica (Li and Wang, 2013) records the origin of the name of Aconite: “(Fuzi) is first planted as aconite, like the head of a crow. The one who is attached to the aconite and born is the Fuzi, like the son attached to the mother.” “Shennong’s Classic of Materia Medica” (Shennong Bencao Jing) was the first to record the taste and efficacy of the Fuzi, and classified it as a lower grade, meaning that it is highly toxic, which also indicates that it has been used in medicine for over a thousand years. The Chinese Pharmacopoeia 2020 (Ch., 2020) summarizes its properties and efficacy as hot, poisonous and returning Yang (阳) to save the rebellion, tonifying fire to help Yang (阳) and dispersing cold to relieve pain. The clinical application of Fuzi is widespread, and one of its main therapeutic effects is cardiac function, which used in the treatment of heart failure, and has been developed into a variety of proprietary Chinese medicines, Shenfu Injection and Qili Qiangxin Capsule are relatively successful examples. Despite the potent cardiotonic effect, Fuzi is also feared for its cardiotoxicity. The purpose of this paper is to illustrate the efficacy and mechanisms of Fuzi in heart failure and other cardiac diseases, as well as cardiotoxicity.

## Phytochemistry

The exploration of the Fuzi began in the 1960s, more than 120 chemical constituents have been isolated and identified from Fuzi (containing aconiti radix), including alkaloids, flavonoids, saponins, sugars and other components. Among these components, mainly alkaloids, including more than 80 kinds of C19-diterpenoid alkaloids, followed by C20-diterpenoid alkaloids, more than 20 kinds, and more than 10 other alkaloids such as amides, quaternary ammonium salts and aporphines, in addition, nearly 20 kinds of non-alkaloid components (Zhou et al., 2015) (Table 1).

**TABLE 1:** The compounds isolated from *A. carmichaelii* Debx.

No.	Chemical component		Source	References
C19-diterpenoid alkaloids				
Non-ester alkaloid	1	Karakoline	Lateral root	Shen et al. (2011)
	2	Senbusine B	Lateral root	Konno et al. (1982)
	3	Senbusine A	Lateral root	Konno et al. (1982)
	4	Neoline	Lateral root, principal root	Shen et al. (2011), Shim et al. (2003)
	5	Fuziline	Lateral root	Shen et al. (2011)
	6	Talatizamine	Lateral root	Shen et al. (2011)
	7	Karakanine	Lateral root	Chen et al. (2003)
Monoester alkaloids	8	*N*-ethylhokbusine B	Lateral root	Xiong et al. (2012b)
	9	(-)-(A-*b*)-14*α*-benzoyloxy-*N*-ethyl-1*α*,8*β*,15*α*-trihydroxy-16*β*,18-dimethoxyaconitane	Lateral root	Jiang et al. (2013)
	10	(-)-(A-*b*)-14*α*-benzoyloxy-*N*-ethyl-1α,8*β*,15*α*-trihydroxy-6*α*,16*β*,18-trimethoxyaconitane	Lateral root	Jiang et al. (2013)
	11	(-)-(A-*b*)-14*α*-cinnamoyloxy-*N*-ethyl-1α,8β,15*α*-trihydroxy-6*α*,16*β*,18-trimethoxyaconitane	Lateral root	Jiang et al. (2013)
	12	14-*O*-acetylneoline	Principal root	Shim et al. (2003)
	13	14-*O*-cinnamoylneoline	Principal root	Shim et al. (2003)
	14	14-*O*-anisoylneoline	Principal root	Shim et al. (2003)
	15	14-*O*-veratroylneoline	Principal root	Shim et al. (2003)
	16	14-acetyltalatizamine	Lateral root	Konno et al. (1982)
	17	(-)-(A-*b*)-14*α*-benzoyloxy-*N*-ethyl-8*β*,15*α*-dihydroxy-1*α*,16*β*,18-trimethoxyaconitane	Lateral root	Jiang et al. (2013)
	18	(-)-(A-*b*)-14*α*-benzoyloxy-*N*-ethyl-6*α*,15*α*-dihydroxy-1*α*,8*β*,16*β*,18-tetramethoxyaconitane	Lateral root	Jiang et al. (2013)
	19	(-)-(A-*b*)-14*α-*benzoyloxy-8β-ethoxy-N-ethyl-6*α*,15*α*-dihydroxy-1*α*,16*β*,18- trimethoxyaconitane	Lateral root	Jiang et al. (2013)
	20	(-)-(A-*b*)-14*α-*benzoyloxy-N- ethyl-6*α*,8β,15*α*- trihydroxy -1*α*,16*β*,18- trimethoxyaconitane	Lateral root	Jiang et al. (2013)
	21	Neojiangyouaconitine	Lateral root	Zhang et al. (1992)
	22	(-)-(A-*b*)-14*α-*benzoyloxy-*N*- ethyl-13*β*,15*α*- dihydroxy -1*α*,6*α*,8*β*,16β,18- pentamethoxyaconitane	Lateral root	Jiang et al. (2013)
	23	(-)-(A-*b*)-14α-benzoyloxy-N- ethyl-8β,13β,15α- trihydroxy -1α,6α,16β,18- trimethoxyaconitane	Lateral root	Shen et al. (2011)
	24	(-)-(A-*b*)-14*α-*benzoyloxy-N- ethyl-8β,13β- dihydroxy -1*α*,6*α*,16*β*,18- tetramethoxyaconitane	Lateral root	Jiang et al. (2013)
	25	14-benzoylaconine	Principal root	Zhang et al. (1982)
	26	(-)-(A-*b*)-14*α-*benzoyloxy-N- ethyl-3α,8β,13*β*,15*α*- tetrahydroxy -1*α*,6*α*,16*β*,18- tetramethoxyaconitane	Principal root	Jiang et al. (2013)
	27	(-)-(A-*b*)-14*α-*benzoyloxy-*N*- ethyl-3α,10β,13β,15*α*- tetrahydroxy -1*α*,6*α*, 8*β*,16*β*,18- tetramethoxyaconitane	Principal root	Jiang et al. (2013)
	28	hokbusine A	Lateral root, principal root	Hikino et al. (1983), Hang et al. (1988)
	29	14-benzoylmesaconine	Lateral root	Kitagawa et al. (1984a)
	30	(-)-(A-*b*)-14*α-*benzoyloxy- 3*α*, 8*β*,10*β*,13*β*,15*α*- pentahydroxy -1*α*,6*α*, 16*β*,18- tetramethoxy-*N*-methylaconitane	Lateral root	Jiang et al. (2013)
	31	(-)-(A-*b*)-14*α-*benzoyloxy- 3*α*, 10*β*,13*β*,15*α*- tetrahydroxy -1*α*,6*α*, 8*β*,16*β*,18- pentamethoxy-*N*-methylaconitane	Lateral root	Hikino et al. (1983)
	32	(-)-(A-*c*)-14*α-*benzoyloxy- 3*α*, 10*β*,13*β*,15*α*- tetrahydroxy -1*α*,6*α*, 8*β*,16*β*,18- pentamethoxy-*N*-methylaconitane	Lateral root	Jiang et al. (2013)
	33	Hokbusine B	Principal root	Hikino et al. (1983)
Diester alkaloids	34	Crassicauline A	Principal root	Shim et al. (2003)
	35	Aconitine	Lateral root	Shen et al. (2011)
	36	(-)-(A-b)-8*β*-acetoxy-14*α*-benzoyloxy-*N*-ethyl-13*β*,15*α*-dihydroxy-1*α,*6*α,*16*β,*18-tetramethoxyaconitane	Lateral root	Jiang et al. (2013)
	37	(-)-(A-b)- 8*β*-acetoxy-14*α*-benzoyloxy-*N*- ethyl-3*α*, *10β*,13*β*, 15*α*-tetrahydroxy-1*α*, 6*α,16β*, 18-tetramethoxyaconitane	Lateral root	Jiang et al. (2013)
	38	(-)-(A-b)- 8*β*-acetoxy-14*α*-benzoyloxy-*N*- ethyl-3*α*, *10β*,13*β*-trihydroxy-1*α*, 6*α,16β*, 18-tetramethoxyaconitane	Lateral root	Jiang et al. (2013)
	39	Foresaconitine	Principal root	Kitagawa et al. (1984b)
	40	(-)-(A-b)- 8*β*-acetoxy-14*α*-benzoyloxy-*N*- ethyl-15*α*-hydroxy-1*α*, 6*α,16β*, 18-tetramethoxyaconitane	Lateral root	Jiang et al. (2013)
	41	(-)-(A-c)- 8*β*-acetoxy-14*α*-benzoyloxy-*N*- ethyl-13*β*,15*α*-dihydroxy-1*α*, 6*α,16β*, 18-tetramethoxy-19-oxo-aconitane	Lateral root	Jiang et al. (2013)
	42	Hypaconitine	Lateral root	Shen et al. (2011)
	43	(-)-(A-b)- 8*β*-acetoxy-14*α*-benzoyloxy-10*β*, 13*β*,15*α*-trihydroxy-1*α*, 6*α,16β*, 18-tetramethoxy-*N*-methylaconitane	Lateral root	Jiang et al. (2013)
	44	Mesaconitine	Lateral root	Shen et al. (2011)
	45	(-)-(A-b)- 8β,14*α*-benzoyloxy-*N*-ethyl-3*α,13β*, 15*α*-trihydroxy-1*α*, 6*α,16β*, 18-tetramethoxyaconitane	Lateral root	Jiang et al. (2013)
	46	Beiwutine	Principal root	Hang et al. (1988)
	47	Isodelphinine	Lateral root	Zhang et al. (1982)
	48	Aldohypaconitine	Lateral root	Wang et al. (1994)
C20-diterpenoid alkaloids				
Atisine	49	Aconicarmine	Lateral root	Xiong et al. (2012b)
	50	Aconicarchamine B	Lateral root	Jiang et al. (2013)
Hetisine	51	Hetisine	Lateral root	Zhang et al. (2010)
	52	(+)-(13R,19S)-1*β*,11*α*-diacetoxy-2-benzoyloxy-13,19-dihydroxyhetisan	Lateral root	Jiang et al. (2013)
	53	(-)-(13R,19S)- 11*α*,19-dihydroxy-*N*-methyl-13-(S-2-methylbutyryloxy)-2*α*-propionyloxyhetisanium hydroxide	Lateral root	Jiang et al. (2013)
	54	(-)-(13R,19S)- 7*β*, 11*α*,19-trihydroxy-*N*-methl-13-(S-2-methylbutyryloxy)- 2*α*- propionyloxyhetisanium hydroxide	Lateral root	Jiang et al. (2013)
	55	(+)-(13R,19S)-2*α*-isobutyryloxy-7*β*, 11*α*,19-trihydroxy - *N*-methyl-13-(S-2-methylbutyryloxy)hetisanium hydroxide	Lateral root	Jiang et al. (2013)
Napelline	56	Songorine	Lateral root	Shen et al. (2011)
Lycoctine	57	Aconicarchamine A	Lateral root	Shen et al. (2011)
Other alkaloids				
	58	Uracil	Principal root, lateral root	Guo et al. (2013), Han et al. (1997)
	59	Uridine	Principal root	Guo et al. (2013)
	60	Nicotinamide	Principal root	Guo et al. (2013)
	61	6-hydroxymethyl-3-pyridinol	Lateral root	Lei et al. (2013)
	62	Hypoxanthine	Principal root	Guo et al. (2013)
	63	Adenosine	Principal root, lateral root	Guo et al. (2013), He et al. (2013)
	64	5-hydroxymethyl-pyrrole-2-carbaldehyde	Lateral root	Xiong et al. (2012b)
	65	Aconicaramide	Lateral root	Xiong et al. (2012b)
	66	Aconicarpyrazine A	Principal root	Guo et al. (2013)
	67	Aconicarpyrazine B	Principal root	Guo et al. (2013)
	68	Oleracein E	Lateral root	Xiong et al. (2012b)
	69	*N*-(2′-*β*-D-glucopyranosyl-5′-hydroxysalicyl)-4-hydroxy-3-methoxyanthranilic acid methyl ester	Lateral root	Guo et al. (2013)
	70	*N*-(2′-*β*-D-glucopyranosyl-5′-hydroxysalicyl)-4-hydroxyanthranilic acid methyl ester	Lateral root	Guo et al. (2013)
	71	(2*S*,3*S*,4*R*,8*E*)-2-[(2′*R*)- 2′-hydroxylignoceroylamino]-8(E)-octadecene-1,3,4-triol	Principal root	Chinese Medical Sciences (2013)
Other compounds				
	72	FI	Lateral root	Ruan et al. (2000)
	73	FPS-1	Lateral root	Zhao et al. (2006)
	74	Benzoic acid	Lateral root	Lei et al. (2013)
	75	Liquiritigenin	Principal root	Shim et al. (2005)
	76	Isoliquiritigenin	Principal root	Shim et al. (2005)
	77	Liquiritin	Principal root	Shim et al. (2005)
	78	6″-*O*-acetylliquiritin	Principal root	Shim et al. (2005)
	79	(*Z*)-p-coumaric acid 4-O-*β*-glucoside	Lateral root	He et al. (2013)
	80	(*Z*)-feruloyl-4-*β*-glucoside	Lateral root	He et al. (2013)
	81	(*E*)-feruloyl-4-*β*-glucoside	Lateral root	He et al. (2013)
	82	Isomaltol-glucoside	Lateral root	He et al. (2013)
	83	Fuzinoside	Lateral root	Hou (2005)
	84	Gracillin	Principal root	Shim et al. (2005)

**FIGURE 1:**
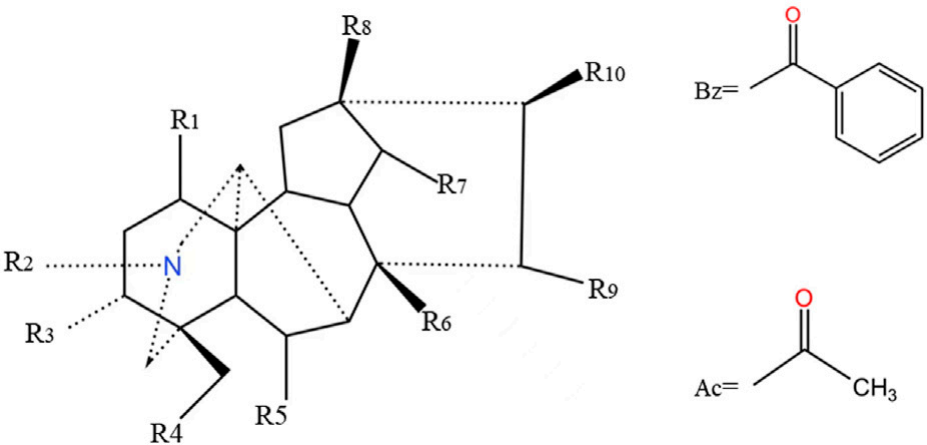
Mother nucleus of C19-diterpenoid alkaloids.

**FIGURE 2:**
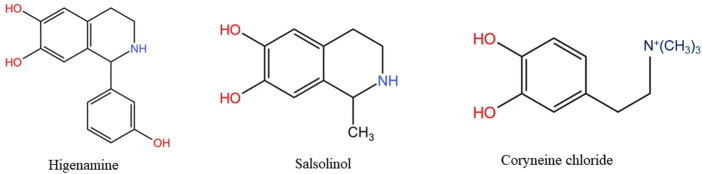
Structural formulae of some water-soluble alkaloids in Fuzi.

C19-diterpenoid alkaloids are the most isolated components from Fuzi and the most toxic components, mainly composed of aconitine skeleton (Figure 1). If different functional groups are connected at positions C-1, C-3, C-6, C-8, C-13, C-14, C-15, C-16, C-18, and C-20 in the parent nucleus, different chemical structures can be displayed (Table 2), thus showing different chemical properties and pharmacological effects (Tang et al., 2017). For example, in the analgesic effect, the ester group at C-8 and C-14 has a greater influence, and in the effect of local anesthesia, the aryl group connected at C-14 is the most important factor affecting its activity, followed by the C-4 group (Bello-Ramírez and Nava-Ocampo, 2004). The acetyloxy group linked to C-8 plays an important role in anti-inflammatory activity (Ren et al., 2010). Similarly, in the structure-activity relationship of toxicity, the change of ester groups at C-8 and C-14 positions also has a great effect on toxicity, but the toxicity is greatly reduced when one ester group is transformed into a monoester compound after hydrolysis (Xiong et al., 2017). Therefore, it can be divided into diester-type, monoester-type and alkanol amine alkaloids, depending on the ester bond contained. For example, 30 diester-diterpenoid alkaloids, such as aconitine, mesaconitine and hypaconitine. 27 monoester-diterpenoid alkaloids, such as benzoylaconine, benzoylmesaconitine and benzoylhypacoitine. And 21 amine alcohol type C19-diterpenoid alkaloids, such as neoline, fuziline (Zhang et al., 2020). Additionally, most of the diester and monoester type alkaloids can be further hydrolyzed into water-soluble alkaloids, so most of them belong to alkanol amine alkaloids, and in addition to the above-mentioned parts, there are also higenamine, salsolinol and coryneine chloride (Figure 2), which have obvious cardiotonic and anti-inflammatory effects although their content is low (Xu et al., 2021a).

**TABLE 2:** Names and substituents of some C19-diterpene alkaloids.

Chemical component	R_1_	R_2_	R_3_	R_4_	R_5_	R_6_	R_7_	R_8_	R_9_	R_10_	References
Aconitine	OCH_3_	C_2_H_5_	OH	OCH_3_	OMe	OAc	H	OBz	OH	OH	Tang et al. (2017)
Hypaconitine	OCH_3_	CH_3_	H	OCH_3_	OMe	OAc	H	OBz	OH	OH	Tang et al. (2017)
Mesaconitine	OCH_3_	CH_3_	OH	OCH_3_	OMe	OAc	H	OBz	OH	OH	Tang et al. (2017)
Benzoylaconine,	OCH_3_	C_2_H_5_	H	H	OMe	H	OH	H	OBz	OH	Zhang et al. (2015)
Benzoylhypacoitine	OCH_3_	C_2_H_5_	H	H	OMe	OCH_3_	OH	H	OBz	OH	Zhang et al. (2015)
Benzoylmesaconitine	OCH_3_	C_2_H_5_	H	H	OCH_3_	OMe	OH	H	OBz	OH	Zhang et al. (2015)
Neoline	OH	C_2_H_5_	H	OCH_3_	OCH_3_	OH	OH	H	H	OCH_3_	Tang et al. (2017)
Fuziline	OH	C_2_H_5_	H	OCH_3_	OCH_3_	OH	OH	H	OH	OCH_3_	Tang et al. (2017)

## Molecular mechanism of Fuzi in the treatment of heart failure

**FIGURE 3:**
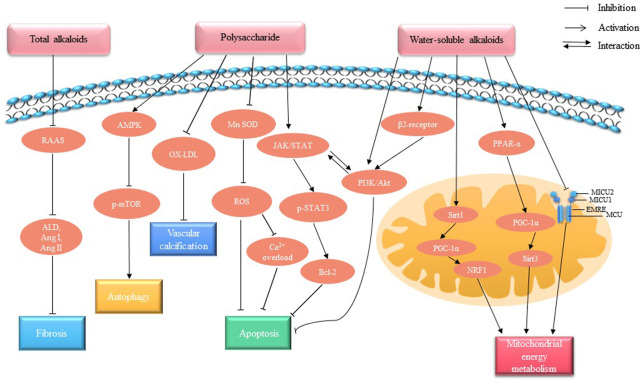
Molecular mechanisms of the cardiotonic effect of Fuzi. Total alkaloids, polysaccharide and water-soluble alkaloids of Fuzi are the main components of cardiac activity. Total alkaloids inhibit myocardial fibrosis mainly by regulating the RAAS system. Polysaccharide promote autophagy and inhibit vascular calcification and apoptosis by regulating AMPK, JAK/STAT pathways and exerting antioxidant effects. Water-soluble alkaloids regulate PI3K/AKT signaling pathway to inhibit apoptosis, and regulate mitochondria-mediated pathways to promote mitochondrial energy metabolism.

Fuzi is known as the best botanical drug of restoring Yang (阳) for resuscitation, and its effect of “restore Yang (阳) and rescue patient from collapse and tonify fire to help Yang (阳)” is explained by modern medicine as “strengthening the heart.” So that in addition to analgesia, it is often used in the treatment of cardiac arrhythmia, myocardial infarction, heart failure and other heart diseases. As for the bioactive components of Fuzi in treating heart failure, there are evidences that total alkaloids (Ye et al., 2013a), water-soluble alkaloids, (Xie, 2012) and polysaccharide (Li et al., 2021) are the main effective substances, which can improve energy metabolism, inhibit peroxide, optimize left ventricular diastolic function and other cardiac protective effects[(Liu et al., 2012a), (Zhao et al., 2012)](Figure 3).

### Total alkaloids

The total alkaloids in Fuzi include diester-diterpenoid alkaloids, monoester-diterpenoid alkaloids and alcohol amine alkaloids. After compatibility and decoction, the diester type alkaloids of toxic components can be converted to the monoester form, and it can be further converted into alcohol amine type alkaloids after absorption by human stomach and intestinal mucosa, thus reducing the toxicity (Li et al., 2016a), (Kuang et al., 2014).

In order to explain which components of Fuzi have cardiotonic effect, Chen et al. (2019a) gave different Fuzi components in the rat model of acute heart failure, such as total alkaloids, water-soluble alkaloids and polysaccharide, etc. The results showed that 10 g crude drug g/kg total alkaloids had the strongest effects on enhancing heart frequency, increasing left ventricular maximum rise rate (+dp/dtmax) and decreasing left ventricular maximum fall rate (−dp/dtmax), at the same time, it could also reduce the Ang I and Ang II and regulating the RAAS system, and ultimately ventricular remodeling can be improved. In China, there is a saying that “Fuzi exhibiting fewer hot characteristics without rhizoma zingiberis,” which is not only because rhizoma zingiberis can promote the dissolution of total alkaloids in Fuzi, so as to play a better role in strengthening the heart, but can also reduce the content of ester components in Fuzi, so as to inhibit its toxicity (Ye et al., 2013b). For example, the combination of total alkaloids and dried ginger extract at 2:1 can enhance the myocardial contractile function and reduce toxic effects in rats with heart failure (Zeng et al., 2011a). Further studies on the compatibility of total alkaloids with rhizoma zingiberis showed that the herbal pair might act as an aldosterone antagonist when acting as angiotensin-converting enzyme inhibitor I (ACE I), thereby regulating RAAS system to reduce Ang II production and to inhibit myocardial fibrosis (Zeng et al., 2011b). Moreover, the total alkaloids can regulate the expression of various ischemic myocardial proteins, which are involved in energy metabolism, signal transduction and antioxidant damage of cardiomyocytes, and thus producing protective effects on ischemic myocardium (Li et al., 2008).

It is worth mentioning that the total alkaloids of Fuzi contain many chemical components. At low doses, it has positive effects on cardiac function and isolated hearts, but with the increase of concentration, it shows cardiac toxicity (Sheng et al., 2019). It can be seen that within a certain dose range, the heart-strengthening effect of Fuzi increases with the increase of dose. However, with the continuous increase of dose, this effect will not increase indefinitely, but the toxic reaction is obvious (Lin et al., 2016; Deng et al., 2010). Here are relatively few researches on the cardiotonic effect of total alkaloids. The reason may be that various chemical components contained in total alkaloids will affect the accuracy of experimental results and doses are an important factor to consider.

### Polysaccharide

Fuzi polysaccharide (FPS), a water-soluble polysaccharide with hydroxyl group of reduced hemiacetals, has antioxidant, apoptosis inhibiting and autophagic activity enhancing effects.

In an early study, it found that FPS could not only reduce liver ischemia-reperfusion injury through its antioxidant effect, but also showed the same protective effect in cardiomyocytes (Lin et al., 2009). Similarly, FPS exhibited protective effects in cardiac myocytes. *In vitro* myocardial ischemia-reperfusion injury cell model, post-treatment with FPS inhibited apoptosis by enhancing the expression of manganese superoxide dismutase (Mn SOD) activity as well as gene expression, promoting the expression of the anti-apoptotic gene Bcl-2, and scavenging excess oxygen radicals produced by mitochondria (Liu and Ji, 2011). In-depth studies revealed that the anti-oxidative radical generation effect of FPS could antagonize calcium overload, reduce intracellular calcium ion concentration, and decrease cardiomyocyte apoptosis (Liu et al., 2012b). In the investigation of its anti-apoptotic mechanism, it was found that FPS can induce the phosphorylation of signal transducers and activators of transcription 3 (STAT3), which is a substrate of JAK-STAT pathway and can promote Bcl-2 expression, exerting cardioprotective effects (Boengler et al., 2008; Liu et al., 2012c).

It is believed that cells recycle amino acids and fatty acids for energy production through autophagy, and that autophagy also helps to remove damaged organelles, thus autophagy plays an important role in the survival of cells (Matsui et al., 2007). And some previous researches have demonstrated that autophagy was upregulated in cardiovascular diseases (Sciarretta et al., 2018). Liao et al. (2013) found that autophagy promotes the survival of cardiomyocytes, and that activated AMPK inhibits mTOR phosphorylation and ultimately promotes cellular autophagy in the FPS-mediated starvation assay, thereby attenuating the cytotoxicity of H9C2 cells caused by starvation and restoring the viability of cardiomyocytes. On this basis, they also found that FPS could weaken the reduction of cellular autophagy caused by oxidatively modified low-density lipoprotein (OX-LDL), thus improving OX-LDL-induced vascular calcification in vascular smooth muscle cells (Liao et al., 2018). Vascular calcification is an important contributor to arterial disease (such as atherosclerosis) (Durham et al., 2018), and these findings suggest that FPS have the potential to treat heart diseases.

Although FPS has cardioprotective effect, the result showed the weakest therapeutic effect of FPS compared to total alkaloids of Fuzi and Fuzi extracts, suggesting that alkaloid components are more important in cardioprotective effect (Wu et al., 2019). This does not mean that FPS has no research value. Compared with alkaloids with toxic effects, it has no obvious cardiac and embryonic toxicity (Ding et al., 2019), which is superior to other components.

### Water-soluble alkaloids

Integrating the previous studies on the treatment of heart diseases with the water-soluble alkaloids in Fuzi, its cardiotonic effect is mainly manifested in anti-cardiac hypertrophy, anti-fibrosis, improving hemodynamics and reducing cell apoptosis (Xu et al., 2021b; (He et al., 2014b). After decades of research, it is generally accepted that higenamine, salsoline and coryneine chloride are the major bioactive components for enhance cardiac function (Wang et al., 2014).

During the period of myocardial ischemia-reperfusion injury, apoptosis of cardiomyocytes aggravates the development of ischemic heart injury and heart failure (Herr et al., 2020). Earlier studies found that the administration of higenamine before rats underwent ischemia-reperfusion injury significantly decreased cytochrome C release and caspase-3 activity and Bax expression, and upregulated Bcl-2 expression, presumably the anti-apoptotic effect of higenamine was related to Bcl-2/Bax (Lee et al., 2006). Chen et al. (2013) further treated cardiomyocytes with doxorubicin, which caused oxidative stress in cardiomyocytes and reduced the content of endogenous antioxidants, and finally activated the mitochondrial apoptosis pathway. However, they found that the combination of higenamine and 6-gingerol could inhibit this pathway, and this inhibition could be relieved by PI3K inhibitor, thus hypothesizing that this inhibition could exert cardiac protective effect through the activation of PI3K/AKT signaling pathway. A further in-depth work revealed that higenamine antagonized apoptosis and protected against ischemia/reperfusion-induced myocardial infarction in rat cardiomyocytes by activating β2-adrenergic receptors, and found that activation of the β2-AR/PI3K/AKT cascade was the key path to its anti-apoptotic effect (Wu et al., 2016). And the structure of higenamine is similar to catecholamines, which explains its excitatory effect on *β*-Adrenergic receptors (Chen et al., 2019b). Interestingly, recent trials demonstrated that higenamine is also an α1-adrenergic receptor antagonist, which helped to lower blood pressure and inhibit platelet aggregation (Zhang et al., 2019a). Regarding the PI3K-AKT signaling pathway, Chinese scholars also found that activation of this pathway may be one of the molecular mechanisms for its therapeutic effect in the treatment of acute heart failure. And since the JAK-STAT pathway can interact with the PI3K-AKT pathway (Lu et al., 2008), it is speculated that this pathway is among the mechanisms of action of Fuzi in reducing cardiomyocyte apoptosis and protecting the heart (Wang et al., 2019).

In recent years, the relationship between mitochondria and cardiomyocyte energy metabolism and heart failure has gradually become a hot topic in clinical research. And abnormal mitochondrial function and cellular metabolism disorders often affect myocardial systolic dysfunction and left ventricular remodeling (Lopaschuk et al., 2021). LKB1/AMPK/Sirt1 are important targets of adriamycin-induced myocardial injury, and these targets also assume essential roles in mitochondrial energy metabolism (Cantó and Auwerx, 2009). The combination of higenamine and 6-gingerol was reported to promote the expression of mRNA and protein of LKB1, AMPKα1, and Sirt1, which could alleviate the disturbance of myocardial mitochondrial energy metabolism (Wen et al., 2020). In addition, on the basis of clarifying that the combination of Fuzi and rhizoma zingiberis can promote mitochondrial biosynthesis through the Sirt1/PGC-1α pathway and regulate mitochondrial energy metabolism through the PPARα/PGC-1α/Sirt3 signaling pathway (Lu et al., 2017; Wen et al., 2019a). Wen et al. (2019b) demonstrated that salsoline could reduce adriamycin-induced myocardial cell injury and apoptosis to varying degrees, mainly by inhibiting the over-activation of MCU (mitochondrial calcium uniporter) signaling pathway, thereby reducing mRNA and protein expressions of MCU, MICU1 (mitochondrial calcium uptake 1) and MICU2 (mitochondrial calcium uptake 2), thus improving mitochondrial energy metabolism and cardiac function. MCU plays an important role in cardiovascular diseases because it binds to MICU1, MICU2, and EMRE (essential MCU regulator) proteins to control Ca^2+^ transport and maintain intracellular calcium homeostasis (Mallilankaraman et al., 2012).

A final point to mention is the cardiotonic function of coryneine chloride. Previous work clearly showed that coryneine chloride significantly increased the contraction amplitude and frequency of the isolated guinea pig right atrium, and in combination with atropine, the contraction frequency and amplitude of the atrium increased by 215% and 415% (Zhou, 2011). It is worth mentioning that although the cardiotonic effect of coryneine chloride is obvious, its cardiotonic mechanism has not been reported in recent years. And there is still controversy about whether the water-soluble alkaloids of neoline and fuziline have cardiotonic effects. Modern pharmacological studies showed that they have pharmacological activities such as cardiotonic, anti-inflammatory and analgesic (Li et al., 2014a; Khan et al., 2018). For example, (Xiong et al., 2012a) *in vitro* experimental studies showed that fuziline and neoline significantly restored cardiac rhythm and improved cell viability in a pentobarbital sodium-induced cardiomyocyte injury model. However, by constructing rat model of acute heart failure, Zhang et al. (2019b) found that fuziline and neoline did not show cardiotonic effect in acute rat model of he. It is also important to note that Fuzi has very little water-soluble alkaloid content, which will be lost in the processing process. Therefore, Zhang et al. (2019b) concluded that higenamine, salsoline are the key quality markers of raw Fuzi for cardiac activity, while salsoline is the key quality marker of processed products of Fuzi for cardiac activity. It can be seen that although the water-soluble alkaloids of Fuzi have clear cardiotonic effect, fewer components were found, and the molecular mechanism of some components related to cardiac-strengthening is not well studied, so it is necessary to further research.

## Molecular mechanism of cardiotoxicity in Fuzi

Fuzi is commonly used in the treatment of heart failure, but its toxicity is undeniable. The contradiction between the “toxicity” and “effectiveness” of Fuzi has been discussed by different medical practitioners throughout the ages. Some experts believe that the toxicity of Fuzi makes it necessary to be used in low doses. However, the School of the Fire God, which inherited and carried forward Zhang Zhongjing’s “warming and supporting Yang (阳) Qi” method, believes that the use of Fuzi must be used in large doses in order to be sufficiently effective in saving patients with acute illnesses. Therefore, modern masters of traditional Chinese medicine evaluated Fuzi as “the most useful and most difficult medicine” (Li and Peng, 2017). A search of the Chinese domestic paper databases uncovered that a total of 508 cases involving adverse reactions to Fuzi were reported between 1953 and 2017, and most of them occurred 3 h after drug administration (Yang et al., 2017a). The mechanism of Fuzi toxicity involves the nervous system, digestive system and cardiovascular system. Heart as one of the most important target organs of Fuzi toxicity, and clinical poisoning is mainly manifested by symptoms such as cardiac arrhythmia, ventricular tachycardia and even cardiac arrest (Yang et al., 2018). Aconitine, a highly toxic component of Fuzi, can cause poisoning in adults at 0.2 mg orally and can be fatal at 2–4 mg, which makes Fuzi contraindicated in clinical utilization. For example, the dosage of Fuzi is regulated in Asian countries such as Korea and Japan. And Chinese food and drug regulations clearly state that aconitum-like toxic botanical drugs cannot be used as raw materials for food processing, and the Chinese Pharmacopoeia 2020 clearly specifies the clinical use of Fuzi at a dose of 3–15 g/d, and it needs to be decocted first or for a longtime during decoction (Ch., 2020).

The main components that cause cardiotoxicity are aconitine, hypaconitine and mesaconitine, which belong to the C19-diester-diterpenoid alkaloids (Nyirimigabo et al., 2015), and aconitine has been the most frequently studied. The zebrafish model showed no significant differences in the pharmacokinetics of aconitine and mesaconitine, and zebrafish embryos placed in mesaconitine showed similar cardiotoxicity to aconitine (Liu et al., 2019). At present, the mechanisms of cardiotoxicity of Fuzi are mainly focused on intracellular Ion overload, lipid peroxidation, and apoptosis. In prior it was demonstrated that aconitine promotes apoptosis in H9C2 cells and rat ventricular myocytes (Nie et al., 2017). In terms of Ion overload, Zhou et al. (2013) demonstrated that 1 μM aconitine accelerated the activation of L-type calcium channel and prolonged action potential duration in rat ventricular myocytes, and that 5 μM and 10 μM aconitine induced arrhythmias by triggering activity and late post-depolarization; further insight revealed that aconitine not only deactivates open sodium channels, leading to sodium inward flow, but also activates L-type calcium channels by sustained activation of sodium channels, causing calcium overload and arrhythmia. At the same time, calcium overload also activates phosphorylated p38 *via* the p38-MAPK pathway, and stimulates p53 expression and activates caspase-3, all of which together promote apoptosis in ventricular myocytes (Sun et al., 2014a). Similarly, aconitine induced cardiac dysfunction and apoptosis by modulating calcium signaling pathways in zebrafish and H9C2 cells (Li et al., 2020).

**FIGURE 4:**
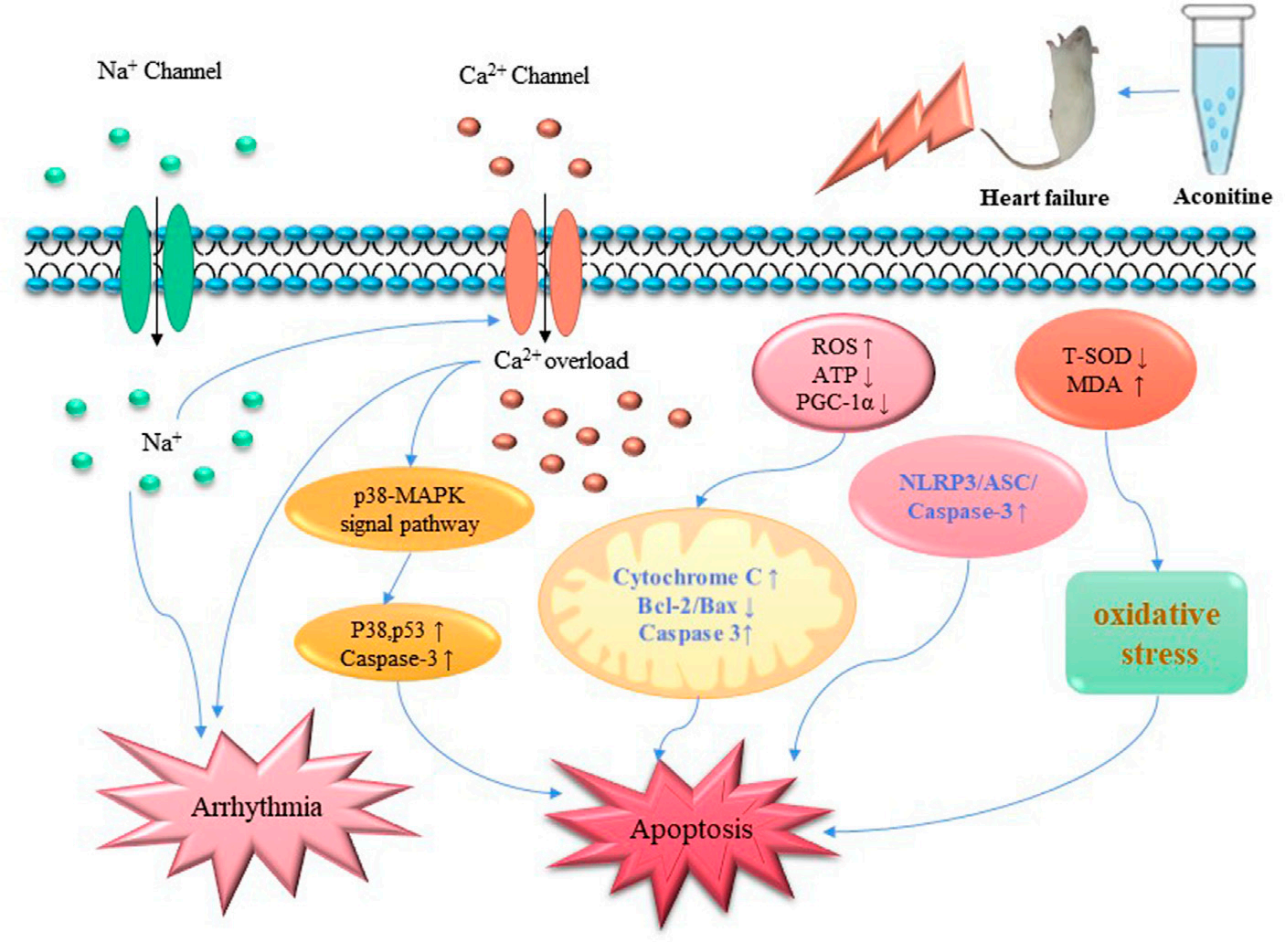
Signaling pathways of Fuzi cardiotoxicity. Aconitine can not only lead to Ion overload, but also increase ROS content, leading to oxidative stress. It also activates mitochondria mediated apoptosis pathway by regulating PGC-1α content. It induces cardiomyocyte inflammation leading to apoptosis by activating NLRP3/ASC/caspase-3 signaling pathway.

Additionally, mitochondria are involved in reactive oxygen species production, oxidative phosphorylation, and metabolic pathways. Apoptotic pathway mediated by mitochondria is currently viewed as one of the major pathways that induce apoptosis (Ding et al., 2012). In the research of mitochondria-mediated apoptosis in H9C2 cells, it was found that aconitine could induce mitochondrial dysfunction by decreasing PGC-1α expression in addition to increasing ROS and decreasing ATP content. The main role of PGC-1α here is to promote mitochondrial biosynthesis. In a further study, cardiomyocytes exposed to aconitine solution also exhibited reduced expression of PGC-1α and a series of changes such as a significant decrease in Bcl-2/Bax ratio and an increase in the levels of cytochrome C and caspase-3, which ultimately led to apoptosis in H9C2 cells (Gao et al., 2018). In addition, Peng et al. (2020) considered that the toxicity of aconitine in cardiomyocytes was mainly related to the activation of TNF-α and NLRP3-inflammasomes pathways. Treatment of zebrafish with aconitine showed a decrease in T-SOD activity and an increase in MDA content, suggesting that aconitine can lead to the occurrence of oxidative stress, while ROS can cause lipid modification, DNA and protein damage leading to apoptosis (Xia et al., 2021) (Figure 4).

Although cardiotoxicity due to the diester diterpenoid alkaloids in Fuzi is most typical, studies showed that Fuzi extracts also have certain cardiotoxic properties. After the action of a certain concentration of water extract of Fuzi on H9C2 cells, it caused the elevation of superoxide and intracellular ROS in mitochondria, which led to the generation of non-specific pore channels (such as mPTP) in the inner mitochondrial membrane, finally, making the outflow of contents such as cytochrome C and thus causing apoptosis, and the elevated ROS also reduced the oxidative phosphorylation function and decreased the production of ATP; in addition, it were found to inhibit the mRNA and protein expression of PGC-1α, an important factor of mitochondrial biosynthesis, suggesting that PGC-1α may be one of the important factors of myocardial injury triggered by water extract of Fuzi (Zhao et al., 2015). The alcohol extract also showed cardiotoxic effect. Alcohol extract of Fuzi increased the levels of leucine and glutamine in rats, and also promoted the expression of PI3K, AKT, and mTOR. mTOR, a downstream factor of PI3K/AKT pathway, either overexpressed or inhibited induced heart diseases (Ananieva et al., 2016), and the result showed that alcohol extract of Fuzi induced apoptosis in cardiomyocytes through activation of PI3K/AKT/mTOR signaling pathway. In addition, the experimental data also demonstrated a significant increase in TGF-β1 expression in heart tissues after the treatment with the alcohol extract, and interestingly, TGF-β1 contributes to the development of cardiac fibrosis, hypertrophy and apoptosis, suggesting that it also produced cardiotoxicity by modulating the TGF-β signaling pathway. At the same time, the results of the study also indicated that the alcohol extract of Fuzi was more poisonous than the water extract (Huang et al., 2018).

The cardiotoxicity of Fuzi is objective, but the specific mechanism of toxicity has not been elucidated in detail, and the current studies have focused on ion channels, oxidative damage and mitochondrial dysfunction and other related pathways. In order to more comprehensively evaluate the toxic and potent effects of Fuzi, it is necessary to scientifically interpret the contents of the botanical drug from six aspects: variety, quality, processing, property, efficacy, application (Peng, 2017). And that is mainly from the following aspects to enhance efficiency and reduce toxicity: 1) Using authentic botanical drug, the herbs from the geo-authentic region are characterized by superior form and efficacy. For example, the abundance of chemical marker such as neoline and songorine was higher in Jiangyou Fuzi in Sichuan Province, and contains lower levels of toxic components such as diester-diterpenoid alkaloids, and higher levels of cardiac components such as salsoline (He et al., 2017). 2) Using a reasonable method of concoction, the degradation of the diester type components in Fuzi to monoester type alkaloids through the concoction process to reduce cardiotoxicity. 3) Rational combination, such as Fuzi with ginseng, ginger, licorice, etc., can not only reduce the content of toxic components, but also increase the therapeutic effect. 4) Extending the decoction time by “first decoction or long decoction” to allow full hydrolysis of the diester-diterpenoid alkaloids. 5) Using the method of syndrome differentiation and treatment, implies the study of toxicity and medicinal effects of Fuzi in combination with different models of disease evidence (He et al., 2015). 6) The dosage is the key factor of drug poisoning. For example, with the increase of dosage, the water extract of Fuzi with cardiotropic effects can cause adverse reactions such as insufficient myocardial contractility and reduced cardiac output in isolated rat heart (Sun et al., 2014b). Thus, it is clear that the study of the relationship between the toxicity and efficacy of Fuzi is not only related to the drug itself, but also requires the elucidation of the scientific relationship from multiple perspectives of “drug-organism-application.”

## Clinical application of Fuzi on cardiotonic effect

The genus *Aconitum* (Ranunculaceae) in the world about 400 kinds of plants, mainly in the northern hemisphere temperate regions, and in China there are about 211 species, which are mainly divided into three subgenera (*Lycoctonum*, *Aconitum*, and *Gymnaconitum*) (Meng et al., 2018). Historically, Fuzi has been widely used in East Asia, such as China, Japan, and Korea. And it is commonly used to treat rheumatoid arthritis, cardiovascular disease, tumors, and other diseases such as gastroenteritis and edema (Wu et al., 2018).

According to the historical records, the first time that the Fuzi was recorded as a great poison in the “Shennong’s Classic of Materia Medica” (Shennong Bencao Jing), However, in the “Treatise on Febrile Diseases” (Shang Han Lun) compiled by Zhang Zhongjing in the Han Dynasty, the prescriptions using Fuzi accounted for 16.8% of the total (Liu et al., 2022). Some of these formulas, Sini Decoction and Zhenwu Decoction, are commonly used to treat heart failure and other diseases. As preparation technology improves by leaps and bounds, some outstanding prescriptions have been developed into proprietary Chinese medicine, such as Shenfu Injection and Qili Qiangxin Capsule (Table 3). By analyzing the Chinese literature from 1984–2018 and exploring the patterns of medication use by prominent TCM practitioners in the treatment of heart failure, it was found that Fuzi was the second most frequently used in prescriptions (Zheng et al., 2020).

**TABLE 3:** Clinical use of Fuzi for cardiotonic effect.

Preparation name	Origin	Compositions	Clinical usage
Shenfu injection	Zheng Ti Lei Yao	Fuzi, *Panax ginseng* C. A. Mey. [Araliaceae; Ginseng radix et rhizoma rubra]. It contains ginsenoside >0.8 mg/ml and aconitine <0.1 mg/ml	For syncope and decompensation caused by *Yang* deficiency; it can also be used for palpitation, asthma, stomach pain, diarrhea and paralysis caused by *Yang* deficiency
Qili Qiangxin capsule	Synopsis of Golden Chamber	*Astragalus membranaceus* (Fisch.) Bunge [Leguminosae; Astragali radix], *Panax ginseng* C. A. Mey. [Araliaceae; Ginseng radix et rhizoma], Fuzi, *Salvia miltiorrhiza* Bunge [Lamiaceae; Salviae miltiorrhizae radix et rhizome], *Descurainia sophia* (L.) Webb ex Prantl [Brassicaceae; Descurainiae semen lepidii semen], *Alisma plantago-aquatica* L. [Alismataceae; Alismatis rhizome], *Polygonatum odoratum* (Mill.) Druce [Asparagaceae; Polygonati odorati rhizome], *Cinnamomum cassia* (L.) D. Don [Lauraceae; Cinnamoni ramulus], *Carthamus tinctorius* L. [Asteraceae; Carthami flos], *Periploca sepium* Bunge [Apocynaceae; Periplocae cortex] and *Citrus reticulata* Blanco [Rutaceae; Citri reticulatae pericarpium]. Each capsule weighs 0.3 g	For mild to moderate congestive heart failure due to coronary heart disease and hypertension
Fuzi Lizhong pills	Formularies of the Bureau of People’s Welfare Pharmacies	Fuzi, Panax ginseng C. A. Mey. [Araliaceae; Ginseng radix et rhizoma], Zingiber officinale Roscoe [Zingiberaceae; Zingiberis rhizoma], G*lycyrrhiza uralensis* Fisch. [Leguminosae; Glycyrrhizae radix et rhizoma praeparata cum melle] and *Atractylodes macrocephala* Koidz. [Asteraceae; Atractylodis macrocephalae Rhizoma]. Each of them is 90 g	For the treatment of gastric and duodenal ulcers, gastrointestinal bleeding, chronic enteritis, heart failure, dysentery, etc.
Zhenwu decoction	Treatise on Febrile Diseases	*Poria cocos* (Schw.) [Polyporaceae; Poria], *Paeonia lactiflora* Pall. [Paeoniaceae; Paeoniae radix alba], *Zingiber officinale* Roscoe [Zingiberaceae; Zingiberis rhizoma]*,* and Fuzi 9 g each, 6 g of *Atractylodes macrocephala* Koidz. [Asteraceae; Atractylodis macrocephalae Rhizoma]	For the treatment of chronic heart failure
Mahuang Xixin Fuzi decoction	Treatise on Febrile Diseases	*Ephedra sinica* Stapf [Ephedraceae; Ephedrae herba] and *Asarum heterotropoides* F. Schmidt [Aristolochiaceae; Asari radix et rhizome] 6 g each, and 15 g of Fuzi	For the treatment of colds, rhinitis, bradyarrhythmia, etc.
Sini decoction	Treatise on Febrile Diseases	300 g of Fuzi, 200 g of *Zingiber officinale* Roscoe [Zingiberaceae; Zingiberis rhizoma] and 300 g of *Glycyrrhiza uralensis* Fisch. [Leguminosae; Glycyrrhizae radix et rhizoma praeparata cum melle]	For the treatment of shock, diarrhea, myocardial infarction, heart failure, etc.
Sini Renshen decoction	Treatise on Febrile Diseases	15 g of Fuzi, 25 g of *Zingiber officinale* Roscoe [Zingiberaceae; Zingiberis rhizoma], 30 g of *Glycyrrhiza uralensis* Fisch. [Leguminosae; Glycyrrhizae radix et rhizoma praeparata cum melle] and 15 g of *Panax ginseng* C. A. Mey. [Araliaceae; Ginseng radix et rhizoma]	Returning *Yang* to rescue the rebellion and benefiting *Qi*
Huiyangjiuji decoction	Tao’s Six Books on Typhoid Fever	9 g of Fuzi, 6 g of *Zingiber officinale* Roscoe [Zingiberaceae; Zingiberis rhizoma], 6 g of *Panax ginseng* C. A. Mey. [Araliaceae; Ginseng radix et rhizoma], 6 g of *Glycyrrhiza uralensis* Fisch. [Leguminosae; Glycyrrhizae radix et rhizoma praeparata cum melle], 9 g of *Atractylodes macrocephala* Koidz. [Asteraceae; Atractylodis macrocephalae Rhizoma], 3 g of *Cinnamomum cassia* (L.) D. Don [Lauraceae; Cinnamomi cortex], 6 g of *Citrus reticulata* Blanco [Rutaceae; Citri reticulatae pericarpium], 3 g of *Schisandra chinensis* (Turcz.) Baill. [Schisandraceae; Schisandrae chinensis fructus], 9 g of *Poria cocos* (Schw.) [Polyporaceae; Poria], 9 g of *Pinellia ternata* (Thunb.) Ten. ex Breitenb. [Araceae; Pinelliae rhizome praeparatum cum zingibere et alumine]	For the treatment of excessive vomiting and diarrhea caused by acute gastroenteritis, shock and heart failure
Baitong decoction	Treatise on Febrile Diseases	15 g of Fuzi, 6 g of *Zingiber officinale* Roscoe [Zingiberaceae; Zingiberis rhizoma] and 4 segments of *Allium fistulosum* L. [Amaryllidaceae; Bulbus Allii Fistulosi]	For the treatment of reducing *Yin* to revive *Yang* and dredging *Qi* between the upper and lower
Xinbao pills	Ministry of Health Pharmaceutical Standards for Prepared Chinese medicine Volume 18	*Datura metel* L. [Solanaceae; Daturaeflos], *Panax ginseng* C. A. Mey. [Araliaceae; Ginseng radix et rhizoma], *Cinnamomum cassia* (L.) D. Don [Lauraceae; Cinnamomi cortex], Fuzi, Cervi Cornu pantotrichum, Borneolum syntheticum, Moschus, *Panax notoginseng* (Burk.) F.H.Chen [Araliaceae; Notoginseng radix et rhizome] and *Bufo bufo gargarizans* Cantor [Bufonidae; Bufonis venenum]. Each pill weighs 60 mg	For the treatment of chronic heart failure caused by heart and kidney *Yang* deficiency; Sinus bradycardia, pathological sinus syndrome, and angina from ischemic heart disease
Liuweihuiyang decoction	Jing Yue’s Collected Works	15 g of *Panax ginseng* C. A. Mey. [Araliaceae; Ginseng radix et rhizoma], 9 of Fuzi, 6 g of *Zingiber officinale* Roscoe [Zingiberaceae; Zingiberis rhizoma], 3 g of *Glycyrrhiza uralensis* Fisch. [Leguminosae; Glycyrrhizae radix et rhizoma praeparata cum melle], 30 g of *Rehmannia glutinosa* (Gaertn.) Libosch. ex Fisch. and C. A. Mey. [Orobanchaceae; Rehmanniae radix] and 9 g of *Angelica sinensis* (Oliv.) Diels [Apiaceae; Angelicae sinensis radix]	For the treatment of coronary heart disease and angina pectoris
Shenfulongmujiuni decoction	Pediatrics of Traditional Chinese Medicine	*Panax ginseng* C. A. Mey. [Araliaceae; Ginseng radix et rhizoma], Fuzi, Drgonsbones, *Paeonia lactiflora* Pall. [Paeoniaceae; Paeoniae radix alba], *Glycyrrhiza uralensis* Fisch. [Leguminosae; Glycyrrhizae radix et rhizoma praeparata cum melle] and Ostrea gigas Thunberg [Ostreidae; Ostreae concha]. Each of them is 15 g	For the treatment of pneumonia, abdominal leakage, heart failure, acute and chronic gastroenteritis with excessive vomiting and leaking, and early symptoms of shock in certain critical illnesses
Poge Jiuxin decoction	Intensive Disease Experience Album	30–300 g of Fuzi, 60 g of *Glycyrrhiza uralensis* Fisch. [Leguminosae; Glycyrrhizae radix et rhizoma praeparata cum melle], 60 g of *Zingiber officinale* Roscoe [Zingiberaceae; Zingiberis rhizoma], 10–30 g of *Panax ginseng* C. A. Mey. [Araliaceae; Ginseng radix et rhizoma rubra], 60–120 g of *Cornus officinalis* Sieb. and Zucc. [Cornaceae; Corni fructus], 30 g of Drgonsbones, 30 g of Ostrea gigas Thunberg [Ostreidae; Ostreae concha] Concha, 30 g of Magnetitum and 0.5 g of Moschus	For all patients with dying heart failure and systemic failure

### Shenfu injection

Shenfu Injection (SFI) is derived from the “Shenfu Decoction,” which was originally published in “Zheng Ti Lei Yao” by Xue Ji in the Ming Dynasty (Xu et al., 2020), containing mainly two Chinese botanical drugs, Ginseng and Fuzi, with ginsenosides, alkaloids and aconitine derivatives as its main ingredients (Li et al., 2016b). SFI is a Chinese medicine preparation based on this and combined with modern preparation technology (official approval code: certification number Z20043117; China Ya’an No. 110804). It has the effect of enhancing coronary blood flow and protecting myocardium and vascular endothelial cells (Li et al., 2014b), so it is widely used in the treatment of anti-heart failure, shock and cardiac arrhythmia (Huang et al., 2019). The therapeutic mechanisms mainly involve anti-apoptosis, inhibition of myocardial fibrosis and ventricular remodeling.

*In vitro* and *in vivo* studies revealed that SFI inhibits apoptosis not only by upregulating the anti-apoptotic protein Bcl-2 and inhibiting the activation of caspase-3, but also by regulating the two major apoptotic pathways of Fas/Fas-L and Bcl-2/Bax, thereby altering the balance of microRNA to inhibit apoptosis (Wang et al., 2009; Yan et al., 2018). Recently, it showed that microRNA can be used as a diagnostic marker for cardiovascular diseases (Tijsen et al., 2012). MEF2A (cardiomyocyte-specific enhancer factor 2A), a target gene of miR-19a-3p, was showed to be essential for energy metabolism in the adult heart in the MEF2A knockdown assay (van Eldik and Passier, 2013). Meanwhile, the MEF2 transcription factor family is an important transcription factor for myocyte hypertrophy (Youn et al., 2000). Mao et al. (2018) first identified that SFI upregulated miR-19a-3p expression, thereby reducing MEF2A mRNA and protein expression and regulating MEF2 signaling pathway-mediated protein expression of *β*-MHC, BNP, and TRPC1, thereby alleviated myocardial hypertrophy. Myocardial fibrosis is closely associated with several heart diseases and can lead to myocardial remodeling and eventually heart failure. The TGF-β/Smads pathway is closely associated with the development of myocardial fibrosis. It was found that SFI treatment significantly reduced the expression of TGF-β1, Smad2 and Smad3 in rats, and conversely, increased the expression level of the inhibitory protein Smad7 and protected the heart (Ni et al., 2017). SFI also improves cardiac function by reducing the secretion of IL-6 as well as TNF-α in rats with heart failure, enhancing myocardial contractility, and improving the hemodynamic disturbance (Fu, 2012). New evidence showed that SFI can produce endothelial-dependent vasodilatory effects through the NO-cGMP pathway, specifically through the PI3K/AKT signaling pathway to upregulate e NOS mRNA and protein expression to increase e NOS content, promote e NOS serine 177 site phosphorylation and inhibit threonine 495 site phosphorylation to enhance e NOS activity, thus promoting the synthesis and release of NO in endothelial cells, to inhibit smooth muscle cell proliferation, platelet aggregation and reduce cardiac pre and post load (Zhu et al., 2020).

### Qili qiangxin capsule

Qili Qiangxin Capsule (QLQX) is a proprietary Chinese medicine made of 11 botanical drugs, including *Astragalus*, *Ginseng*, and Fuzi, which was proved to reduce NT-pro BNP levels and improve left ventricular ejection fraction in patients with chronic heart failure. And it was approved by the Chinese Food and Drug Administration for clinical use in the treatment of chronic heart failure in 2004 (Liang et al., 2016) (State Drug Administration Z20040141), followed by international recognition of the research results on QLQX evidence-based medicine in 2013. Chemical analysis has identified its main components as flavonoids, saponins, diterpenoid alkaloids, etc. (Yun et al., 2018). Pharmacological studies showed that QLQX protects cardiomyocytes and mitochondrial function (Zhang et al., 2013), improves endothelial cell function (Chen et al., 2015), and delays myocardial remodeling (Hou et al., 2019).

In a previous series, QLQX inhibited apoptosis and cardiac remodeling by activating the Neuregulin-1/AKT signaling pathway and inhibiting the p53 pathway in rats with heart failure (Wang et al., 2015). It also improved left ventricular function in rats with heart failure induced by chronic myocardial infarction, reduced interstitial fibrosis by decreasing type I and type III collagen mRNA expression, and protected mitochondrial morphology, these changes may be related to reduced apoptosis and upregulation of VEGF expression, and in which AKT is involved (Liang et al., 2016). The anti-myocardial fibrosis effect of QLQX was also manifested by inhibition of the TGF-β1/Smad3 pathway and promotion of TGF-β3/Smad7 expression of the inhibitory signaling pathway. It also has a regulatory role for miRNAs, as Smad3 mRNA here is a direct target of miR-345-3p regulation. Secondly, it downregulated Bax/Bcl-2, MMP-2 and TIMP-2 levels as a way to attenuate NF-κB-induced cardiac inflammatory response and cardiac fibrosis, which together inhibited adriamycin-induced chronic heart failure (Sun et al., 2020). Zhao et al. (2019) demonstrated for the first time that QLQX protects cardiomyocytes from mitochondria-dependent apoptosis induced by oxidative stress *via* the PI3K/AKT/GSK3β signaling pathway *in vivo* and *in vitro*. Isoprenaline (ISO) is one of the catecholamine adrenergic receptor agonists. Studies showed that isoprenaline can lead to cardiac pathological changes such as cardiac hypertrophy and fibroblast proliferation, and that catecholamines levels are elevated in the progression of heart failure (Wang et al., 2018). The exact evidence confirmed that ISO inhibits the expression of p-AKT, p-mTOR, and conversely, QLQX significantly increases the p-AKT/AKT, p-mTOR/mTOR ratio, and the opposite results occur with AKT inhibitors, these results illustrated that QLQX inhibits ISO-induced myocardial injury by activating the AKT/mTOR signaling pathway (Fan et al., 2019). A comprehensive study by pharmacokinetics, network analysis and experimental validation revealed that QLQX significantly reduced IL-6, p-JAK2, and p-STAT3 levels while increasing VEGFA levels to repair homocysteine-induced microvascular endothelial injury, suggesting that it could inhibit inflammatory processes in heart failure and promote angiogenesis in chronic heart failure through the JAK/STAT signaling pathway (Zhang et al., 2019c).

### Sini decoction

Sini Decoction is a recipe which consists of Fuzi, *Rhizoma Zingiberis*, and liquorice in the ratio of 3:2:3, was first recorded in Zhang Zhongjing‘s “Treatise on Febrile Diseases,” and has been clinically applied with remarkable efficacy in the treatment of cardiovascular diseases (Tan et al., 2018), with various pharmacological effects such as cardiac strengthening, inflammation inhibition, and antioxidant. The anti-heart diseases mechanism of Sini decoction is multifaceted, such as protecting cardiomyocytes, regulating blood pressure and lipids, etc. (Shu et al., 2019).

It was found that Sini decoction can reduce the level of Toll-like receptors in patients and regulate TGF-1 and collagen content in myocardial tissue and serum to a certain extent, which has the potential to improve early ventricular remodeling after myocardial infarction (Liu et al., 2014). It also reduced the occurrence of lipid peroxidation by increasing the levels of Cu-Zn SOD and Mn SOD (Zhao et al., 2005), an antioxidant enzyme in myocardial mitochondria, further protecting mitochondrial membrane integrity, inhibiting caspase-9 and 3 activation and reducing the occurrence of apoptosis in cardiac myocytes, improving cardiac function in multiple ways and ultimately preventing the occurrence of heart failure (Zhao et al., 2010). Modern studies found that the efficacy of Sini decoction in the treatment of myocardial fibrosis. And its mechanism may be related to the inhibition of Rho A/ROCK signaling pathway activation. After Rho A activates the downstream factor ROCK, ROCK 1 in the ROCK family then affects the differentiation of cardiac fibroblasts, and which plays an important role in myocardial fibrosis (Yang and Zhang, 2016). Sini Decoction further upregulated e NOS expression after inhibiting the Rho A/ROCK signaling pathway to increase NO levels while elevating SOD activity to slow oxidative stress (Yang et al., 2017b). In addition, it was hypothesized that the anti-myocardial fibrosis of Sini decoction is also related to the TGF-β1/Smads pathway. Sini decoction not only inhibits the RAAS system to reduce Ang II production, which in turn reduces TGF-β1 mRNA expression (Liao et al., 2010), but also downregulates Smad2 and upregulates Smad7 protein and gene expression (Liao et al., 2012), while TGF-β1 mainly transmits fibrotic signals through Smads protein. One of the receptors for bile acids is nuclear farnesoid X receptor (FXR). Bile acids can further induce the expression of PPARα (peroxisome proliferator–activated receptor α) upon activation of FXR, a negative regulator of the NF-κB signaling pathway, which attenuates lipid peroxidation levels and combats inflammatory damage (Pineda Torra et al., 2003; Michalik and Wahli, 2006). There is no doubt that the NF-κB signaling pathway is a major contributor to the regulation of the inflammatory response in the heart. Zhou et al. (2017) hypothesized by metabolomic studies that the main mechanism of Sini Decoction for isoproterenol-induced myocardial injury may be that it activates FXR and PPARα and then further reduces the NF-κB signaling pathway-mediated inflammatory response, but this hypothesis needs further confirmation. Likewise, Cheng et al. (2018) showed that Sini Decoction reduced the release of the inflammatory factor TNF-α, attenuated the damage to myocardial cells caused by ischemia and hypoxia, and exerted a myocardial protective effect.

The Chinese patent medicines mentioned above mainly involve Fuzi—Ginseng radix et rhizoma, Fuzi—Zingiberis Rhizoma, Fuzi—Glycyrrhizae radix et rhizoma praeparata cum melle and Fuzi—Cinnamomic ramulus, and so on. Among them, the first three herbal pairs can achieve the purpose of enhancing efficacy and reducing toxicity to a large extent, while the combination of Fuzi and Cinnamomic ramulus can increase the anti-inflammatory and analgesic effects, suggesting that the drug may have certain application value in the treatment of arthritis and other diseases. Likewise, in the combination of Fuzi and Astragali radix, astragalus polysaccharide can not only enhance the function of the immune system, but also prevent and treat atherosclerosis, and enhance the therapeutic effect of Fuzi. In addition, through the exploration of Tai et al. (2021), some innovative herbal pairs were found, such as the effect of Fuzi and Ziziphi spinosae semen combination in the treatment of neurological diseases, the effect of Salviae miltiorrhizae radix et rhizoma and Fuzi combination in the treatment of cardiovascular diseases, and the effect of Fuzi in the treatment of cardiovascular diseases was enhanced by Corydalis rhizoma, Cyathulae radix, and Eucommiae cortex. The discovery of these herbal pairs suggests that Fuzi has a broader application, and it is necessary to further explore the specific mechanism of its action. However, it has to be admitted that studies on the pharmacological mechanism and chemical components of Fuzi are mostly focused on itself and related herbal pairs, so there is still a lack of discussion on the main targets and components of specific Chinese patent medicine for diseases. However, most of the Chinese patent medicines related Fuzi are mainly used in Asia. Therefore, in order to expand its clinical application, molecular mechanism and clinical research are essential. In addition, it is also a good method to use the structure-activity relationship of chemical components to further solve the contradiction between toxicity and efficacy of Fuzi.

## Conclusion and perspectives

The heart is the common target organ for Fuzi therapeutic and toxic effects. As reviewed in this paper, it is evident that the main bioactive compounds of Fuzi cardiac strength are total alkaloids, polysaccharide and water-soluble alkaloids, which involve multiple signaling pathways, such as inhibition of apoptosis, antagonism of RAAS system, promotion of cellular autophagy and inhibition of abnormal mitochondrial energy metabolism. In contrast, the mechanisms of cardiotoxicity of the diester-diterpenoid alkaloids in Fuzi includes induction of apoptosis and promotion of intracellular ion-overload. In addition, the three typical herbal preparations for the treatment of heart failure mentioned this article are also involved in the pathways of NF-κB signal-mediated inflammatory response, TGF-β/Smads-mediated fibrosis and microRNA for the treatment of heart diseases. These suggest an important role for the multi-component, multi-targeted combination of therapeutic effects possessed by Fuzi in the treatment of heart failure and other heart diseases.

The earliest record of Fuzi can be traced back to the 2,000-year-old “Shennong Materia Medica.” Later, in “Treatise on Febrile Diseases,” Zhang Zhongjing believed that raw Fuzi should be used for severe patients with Yang (阳) deficiency, and processed Fuzi should be used for mild patients (Liu et al., 2022). In the past decades, people have been constantly aware of the toxin-effect and dose-effect relationships of Fuzi. At the same time, in order to avoid the toxicity, scholars made multi-dimensional evaluation of Fuzi from the perspective of systematic traditional Chinese medicine, so as to achieve the purpose of effect-enhancing and toxicity-reducing. After confirming the pharmacology and chemical composition of Fuzi, its clinical application has been gradually expanded. In recent years, Fuzi has been developed as a cardiotonic, analgesic, and anti-inflammatory, and is extensively used in Asia, including China, Korea, Japan, and India (Tong et al., 2013). In terms of clinical application, concoction products are mostly used, and its water-soluble alkaloids will be partially lost in the water treatment before concoction, which weakens its cardiac strengthening effect. Therefore, for less water-soluble components, it is reasonable to pay more attention to it, for example, how to improve the extraction rate of such alkaloids and how to more comprehensive elucidation of the molecular mechanism are worthy of research topics. There are many components in total alkaloids, which also makes it controversial to clarify the specific components and mechanism of action of total alkaloids. Therefore, it is important to determine the content of diester-diterpenoid alkaloids and monoester-diterpenoid alkaloids in total alkaloids to control toxicity. Fuzi polysaccharide, as a non-toxic chemical component, can be further developed and utilized, which is an interesting topic.

## Glossary

ACEI: angiotensin-converting enzyme inhibitor
AKT: protein kinase B
ALD: aldosterone
AMPK: adenosine mono-phosphate-activated protein kinase
Ang I: angiotensin I
Ang II: angiotensin II
ASC: apoptosis-associated speck-like protein containing a CARD
Bax: BCL2-Associated X
Bcl-2: B cell lymphoma 2
BNP: brain natriuretic peptide
e NOS: endothelial nitric oxide synthases
EMRE: essential MCU regulator
Fas-L: fas ligand
FXR: farnesoid X receptor
GSK3β: glycogen synthase kinase-3β
IL-6: interleukin 6
ISO: isoprenaline
JAK: Janus kinase
LKB1: liver kinase B1
MAPK: mitogen-activated protein kinase
MCU: mitochondrial Ca^2+^ uniporter
MDA: malondialdehyde
MEF2A: cardiomyocyte-specific enhancer factor 2A
MICU1: mitochondrial calcium uptake 1
MICU2: mitochondrial calcium uptake 2
MMP-2: matrix metallopeptidase 2
mPTP: mitochondrial permeability transition pore
mTOR: mammalian target of rapamycin
NF-κB: nuclear factor-kappa B
NLRP3: NOD-like receptor thermal protein domain associated protein 3
OX-LDL: oxidized low-density lipoprotein
PGC-1α: peroxisome proliferator-activated receptor gamma coactivator 1α
PI3K: phosphatidylinositol 3-kinase
PPARα: peroxisome proliferator–activated receptor α
RAAS: renin-angiotensin-aldosterone system
Rho A: ras homolog family member A
ROCK: rho-associated protein kinase
ROS: reactive oxygen species
Sirt: sirtuins
Smad: small mother against decapentaplegic
SOD: superoxide dismutase
STAT: signal transducers and activators of transcription
STAT3: signal transducer and activator of transcription 3
TGF-β: transforming growth factor-β
TIMP-2: tissue inhibitor of metalloproteinases 2
TNF-α: tumor necrosis factor α
TRPC1: transient receptor potential channel 1
*β*-MHC: *β*-major histocompatibility complex
